# Estimating the mechanical cost of transport in human walking with a simple kinematic data-driven mechanical model

**DOI:** 10.1371/journal.pone.0301706

**Published:** 2024-04-16

**Authors:** Parvat Katwal, Suraj Jaiswal, Dezhi Jiang, Lauri Pyrhönen, Jenni Tuomisto, Timo Rantalainen, Arend L. Schwab, Aki Mikkola

**Affiliations:** 1 Department of Mechanical Engineering, LUT University, Lappeenranta, Finland; 2 Faculty of Sport and Health Sciences, University of Jyväskylä, Jyväskylä, Finland; Imperial College London, UNITED KINGDOM

## Abstract

This work utilizes a simplified, streamlined approach to study the mechanical cost of transport in human walking. Utilizing the kinematic motion data of the center of mass, velocities and accelerations are determined using kinematic analysis; the applied force is then obtained using inverse dynamics. We calculate the mechanical cost of transport per step from both synthetic and measured data, using a very simple mechanical model of walking. The approach studied can serve as an informative gait characteristic to monitor rehabilitation in human walking.

## Introduction

People live longer lives, yet facing an increase in chronic illnesses and disabilities [1]. As a result, the demand, and the costs for health care services are expected to rise. Remote treatment and monitoring can reduce these costs and improve the quality of life of the patient. Here we focus on remote monitoring of rehabilitation in human walking.

Remote monitoring in health care needs simple and robust solutions, preferably using mobile devices. One of the first who demonstrated the successful use of mobile sensors was [2] using inertial measurement unit’s (IMU’s) to determine segmental accelerations. After the introduction of the smartphone many applications were introduced using the sensors from the phone to determine spatio-temporal gait parameters, like step length, step time, gait velocity, and cadence [3–6].

The work presented in this paper is part of a larger project in which the goal is to monitor rehabilitation in walking using a simple motion sensor in combination with a simple mechanical model of walking. The gait parameter which we monitor is cost of transport as estimated by the mechanical model fed by the measured data from the motion sensor. Cost of transport is chosen as gait parameter because it has been shown that humans are optimizers, minimizing positive work in walking [7, 8] and that the energy expenditure in walking for impaired subjects is higher than in healthy ones, see f.i. [9].

The goal of this paper is to investigate whether it is possible to predict the mechanical cost of transport in human walking using only the kinematic motion data of the center of mass of the walking human together with a very simple mechanical model of walking.

The novelty of our approach lies in using a single sensor in combination with a simple mechanical model to predict metabolic energy expenditure in walking. Mechanical work has been shown to be a good predictor for the metabolic cost of transport [10–12]. Simple mechanical model have been used successfully in the past to predict energy expenditure in walking and running [12–18].

## Methods

The proposed algorithm to determine the mechanical cost of transport per step, consists of four stages: (i) acquisition of the Center of Mass (CoM) trajectory (ii) determination of velocities and accelerations, (iii) calculation of the forces, and (iv) computation of the cost of transport.

Initially, the CoM position data is obtained either synthetically using kinematic simulations or experimentally using motion capture with Vicon markers. The obtained CoM trajectory is then used as an input to the inverted pendulum model, and kinematic analysis is performed to determine the velocities and accelerations. Inverse dynamics analysis based on Newtonian mechanics is then performed to obtain the forces required to produce the observed motion. Finally, the mechanical work required for the motion, energy loss due to impact, and mechanical cost of transport are calculated based on the forces obtained, taking negative work into account.

### A simple mechanical model of walking

**Fig 1:**
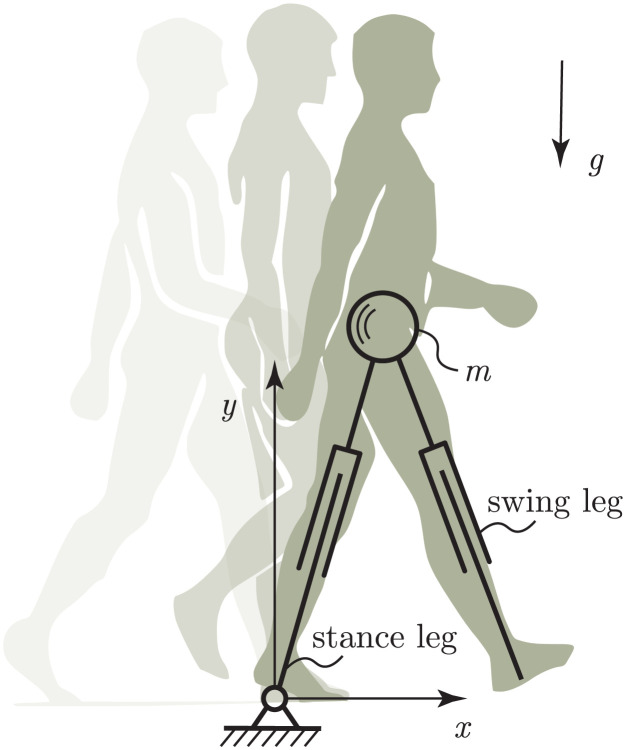
Minimal bipedal walking model. The human body is modelled as a point mass *m*. The two legs are modelled as massless telescopic actuators that can change length and orientation, exert forces, and perform work on the upper body. The walker walks on level ground in a gravity field with field strength *g*. Figure adapted from [20].

One of the simplest dynamical models used to model the human gait cycle is the inverted pendulum model [19]. The inverted pendulum model assumes a human body as a point mass located at the center of mass of the body, while the legs are assumed to be massless. In the inverted pendulum model, the point mass follows a trajectory composed of perfect circle arcs. In human walking, however, the trajectory of the center of mass is not perfectly circular because of changes in leg length during the stance phase. This effect can be incorporated into the inverted pendulum model by introducing an additional degree of freedom where the pendulum is allowed to change its length during the gait cycle (Fig 1).

**Fig 2:**
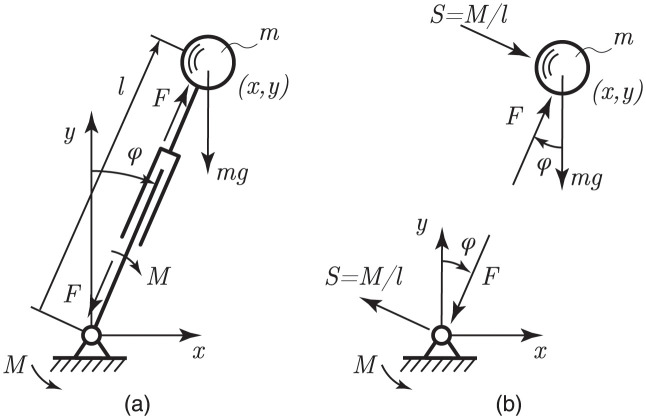
(a) Bipedal walking model. (b) Free body diagram of the point mass together with a diagram of the forces exerted by the model to the support. Figure adapted from [20].

Here we extend the inverted pendulum model, along similar lines as [13], by adding leg extension, see Fig 2. In this way, the center of mass is able to follow an arbitrary trajectory in the *x* − *y* plane. Such a model is comparable to the SLIP model from [18] but with an active spring component.

The advantage of this augmented inverted pendulum model is that it allows an arbitrary point (in planar motion) to be converted into leg angle *φ*, and leg length *l*. When the coordinates are defined such that walking is in the positive x-direction, the origin is attached to the point of contact, and the leg angle from the upright position goes from negative *φ* to positive *φ*. The relationship between Cartesian coordinates and polar coordinates can be written as,

$$\begin{array}{c} \begin{array}{cc} l & =\sqrt{x^{2}+y^{2}},\\ \varphi & =\text{arctan}(\frac{x}{y}). \end{array} \end{array}$$ (1)

From this the velocities of the leg length extension and the pendulum motion can be calculated as,

$$\begin{array}{c} \begin{array}{cc} \dot{l} & =\dot{x}\;\text{sin}\;\varphi +\dot{y}\;\text{cos}\;\varphi ,\\ \dot{\varphi }l & =\dot{x}\;\text{cos}\;\varphi -\dot{y}\;\text{sin}\;\varphi . \end{array} \end{array}$$ (2)

The leg length force *F* and the pendulum motion torque *M* are the constraint forces moving the point mass over the CoM path. As the leg is massless, the pendulum torque *M* can be transformed directly to a shear force *S* = *M*/*l*, acting on the point mass perpendicular to the leg length force. These forces can be determined using the free body diagram shown in Fig 2b as,

$$\begin{array}{c} \begin{array}{cc} F & =m\ddot{x}\;\text{sin}\;\varphi +\left(m\ddot{y}+mg\right)\;\text{cos}\;\varphi ,\\ M/l & =m\ddot{x}\;\text{cos}\;\varphi -\left(m\ddot{y}+mg\right)\;\text{sin}\;\varphi . \end{array} \end{array}$$ (3)

Obviously, the challenge of this model is to determine from the measured CoM kinematic data, the point of foot contact, and the time instance when the previous step ends and the next step starts. In this study, this challenge is overcome by defining the foot placement at midstance, at the maximum of the CoM path, and the change from swing leg to stance leg at the minimum of the CoM path. In this way, a step and the corresponding step-length *d* can be defined from midstance to midstance.

### Energy analysis and cost of transport

**Fig 3:**
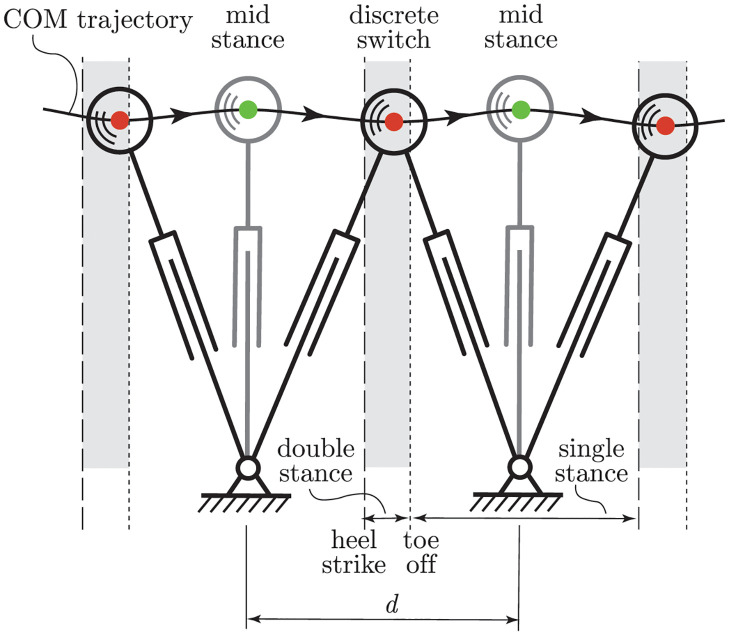
Stance leg switching and double stance phase. The trajectory of the CoM from measured data, where midstance is at the maxima, marked by a green dot, and the discrete switch from swing leg to stance leg is by definition at the minima, marked by a red dot. The double stance phase, which starts with a heel strike and ends at the toe off, is from the measured data, and is indicated by the grey area. The step length *d* is defined from midstance to midstance.

The inverted pendulum model allows classical mechanics to be used for analysis of the energy balance during the human gait cycle. We assume that only one leg at a time is in contact with the ground, and that the change from swing leg to stance leg is instantaneous. Or in other words, that there is no double stance phase in the model. However, in real-life walking, a double stance phase can be observed, as shown in Fig 3. This double stance phase starts with heel strike of the swing leg and ends with the toe-off from the hind leg. During this double stance phase, leg extension forces from both legs are exerted on the CoM. In our model we only have a sequence of single stance phases. However, because our model is kinematic driven, we do have two forces acting on the CoM, one from the leg extension and one from the pendulum motion torque *M*, which acts perpendicular to the leg extension force. In this way we are able to still model the forces and associated power in the double stance phase with our single stance model.

The instantaneous mechanical power output of the bipedal walking model consists of two parts, the leg extension power output, $P_{F}=F\dot{l}$, and the pendulum motion output, $P_{M}=M\dot{\varphi }$. Under the assumption of a periodic walking motion, where the average speed per step is constant, there will be no energy change from one mid-stance to the next. Consequently, the net work of the leg in one step is zero, ∑_*i*=*F*,*M*_ ∫_step_ *P*_*i*_d*t* = ∑_*i*=*F*.*M*_ ∫_step_([*P*_*i*_]^+^ − [*P*_*i*_]^−^)d*t* = 0 with [*P*]^+^ the maximum of the pair (*P*, 0) and [*P*]^−^ the maximum of the pair (−*P*, 0)), and that per step the net positive work equals the net negative work, as in ∑_*i*=*F*,*M*_ ∫_step_[*P*_*i*_]^+^d*t* = ∑_*i*=*F*,*M*_ ∫_step_[*P*_*i*_]^−^d*t*. However, it is known that muscles in combination with tendons are not very proficient at storing work and have to perform negative work for this purpose. Here we follow Srinivasan and Ruina [20] and take this negative work into account by means of a factor *b*. Then the mechanical cost of transport, being the mechanical expenditure per step non-dimensionalized, is then defined as,

$$\begin{array}{c} C=\frac{1}{mgd}\sum_{i=F,M}\int_{0}^{t_{step}}\left({\left[P_{i}\right]}^{+}+b{\left[P_{i}\right]}^{-}\right)dt. \end{array}$$ (4)

For the negative work factor *b* we will follow the seminal work of Abbott [21] and Margaria [22], and take *b* = 0.2.

## Experimental data collection

The walking data was collected from two healthy participants walking indoors at a self-selected pace; Subject 1 is 184 cm tall and weighs 94 kg, while Subject 2 is 172 cm tall and weighs 85 kg. For the data collection, the participants were fitted with 38 retroreflective markers defining the body segments. The marker positions were captured at 200 Hz using a 14-camera optoelectronic motion capture system (Vicon, Oxford, UK).

The motion capture volume consisted of a 6-meter walking area, which included two force plates (AMTI, Watertown, MA, USA) sampling at 1000 Hz. These force plates, which were embedded in the floor, were used to record ground reaction forces during the walking trials. The participants walked barefoot through the motion capture volume at their preferred pace. To ensure constant velocity within the capture volume, 2-meter space were allowed each side of the capture volume for acceleration and deceleration.

After the data collection, the raw marker data were processed using Vicon Nexus software (Vicon, Oxford, UK), and any gaps in the marker trajectories were filled using spline interpolation. The processed marker data was then used to calculate the global position of the center of mass (CoM) of the participants during the walking trials as a weighted mean of the segment CoM trajectories [23]. The body was represented as 11 segments (left foot, left shank, left thigh, right foot, right shank, right thigh, left upper arm, left lower arm and hand, right upper arm, right lower arm and hand, and torso, neck and head) which were identified by the proximal and distal joint centers of each segment. Joint centers were calculated as the midpoint between markers placed mediolaterally (on the ankle, knee, elbow, wrist) or anteroposteriorly (on the shoulder) across the joint. The hip joint centers were determined using markers placed on the anterior superior iliac spines and the posterior edge of the iliac crest, following the model from [24]. The segment mass and center of mass location along the longitudinal axis of the segment were based on the values provided by [23]. The velocities and acceleration of the CoM is obtained through numerical differentiation following a central difference scheme with a step size identical to the sampling frequency of the data. However, due to the presence of inherent noise in the measured data, the calculated velocities and accelerations can be quite noisy. To address this issue, a low-pass filter is applied to the data to attenuate high-frequency noise components and improve the overall quality of the trajectory. The selected filter has a pass band ending at the frequency of 6 Hz and a stop band beginning at the frequency of 10 Hz, effectively reducing noise interference and providing a smoother representation of the CoM motion. The differentiated signals were filtered with the same filter as described above. The global position data of the CoM was the primary focus of this study and served as an input for the kinematic analysis and subsequent stages of the research.

The policy of the Human Sciences Ethics Committee at the University of Jyväskylä where the human experiments were conducted is that Investigations that are non-invasive and are not medical do not require an ethical review in accordance with the local legislation. Based on the policy and consideration of the study design, the decision to undertake the study without a priori ethical review was made by the responsible investigator (TR). The study was conducted in agreement with the Helsinki Declaration. The recruitment period for this study was between February 3 to 6, 2023 and the participants provided verbal consent directly to the experimenter.

## Results

Five different CoM trajectories are analyzed in this paper, three of which are based on synthetic data obtained from mathematical equations to represent pendular, level, and sinusoidal walking. These trajectories serve as a references for comparison with the other two trajectories, which are obtained from measurements using motion capture cameras.

Fig 3 illustrates the step-by-step trajectory of the CoM during human walking, where the solid curve represents the captured CoM trajectory of a human subject. This study employs a simplified mechanical model in which the human leg is depicted as a pendulum, anchored to the ground through a revolute joint. The location of this revolute joint dynamically adjusts to align with the CoM trajectory recorded for each individual step, with the distance between two neighboring revolute joint positions being defined as the step length *d*.

Fig 3 also differentiates between the double and single stance phases of walking. The double stance phase, characterized by both feet making contact with the ground, occurs in the region around the local minimum extremum of the trajectory, marked in grey. Each double stance phase commences with the heel strike of the front foot (indicated by the dashed lines) and concludes with the ‘toe-off’ event of the rear foot (marked by the dotted lines). Conversely, the single stance phase, during which only one foot remains in contact with the ground, occurs in the regions beyond the marked double stance phases.

### Synthetic data

**Fig 4:**
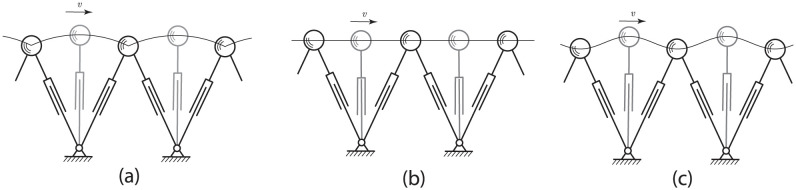
Three synthetic walking motions: (a) pendular, (b) level, and (c) sinusoidal. The configuration at mid stance is drawn in grey, whereas the black configuration is at the discrete switch from swing leg to stance leg.

To be able to interpret the result of the mechanical cost of transport *C* (4) from measured data, we first analyze three extreme gait patterns: pendular, level, and sinusoidal motion. These synthetic gait patterns, illustrating the motion of the CoM, are shown in Fig 4. To ensure a fair and meaningful comparison of the distinct gait patterns, all three synthetic motions share the same parameters: step length *d* = 0.77 m, maximum leg length *l*_0_ = 1.07 m, average velocity *v*_0_ = 1.4 m/s, and mass *m* = 94 kg. For the sinusoidal amplitude we selected the value of *a* = 0.02 m, which is comparable to the vertical motion of the pendular case. The values were chosen to closely represent subject 1’s characteristics.

#### Pendular motion

**Fig 5:**
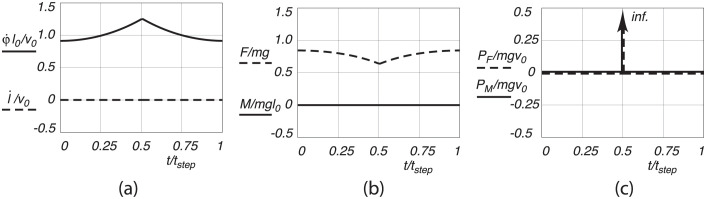
Pendular walking motion, with (a) dimensionless leg extension velocity $\dot{l}/v_{0}$ and pendular velocity $\dot{\varphi }l_{0}/v_{0}$, (b) dimensionless leg extension force *F*/(*mg*) and pendular motion force *M*(*mgl*_0_), and (c) and dimensionless applied leg extension power *P*_*F*_/(*mgv*_0_) and pendular motion power *P*_*M*_/(*mgv*_0_), as a function of dimensionless time *t*/*t*_*step*_, for one step from midstance to midstance.

The pendular motion is characterized by the stance leg acting as an inverted pendulum, leading to a circular arc trajectory of the CoM. In this scenario, the leg acts as a single rigid body. We assume a pure pendular motion under the act of gravity. Starting with an initial velocity at midstance, the point mass accelerates until the swing leg hits the ground and the swing leg makes a discrete switch to stance leg and vice versa, see Fig 5a. At this heel strike an inelastic impact occurs along the new stance leg, reducing the velocity of the point mass. Simultaneously, at toe-off, the hind leg has to apply an impulse to increase and redirect the velocity to the original level such that the walking is cyclic. The energy to increase the speed is equal to the energy lost during impact, because of the cyclic motion assumption. This energy loss per step is *E*_*loss*_ = 1/2*mv*^2^ sin^2^(2*φ*_0_), with *v* the magnitude of the velocity of the CoM just before impact, and *φ*_0_ the angle of the leg at heel strike with respect to the vertical [19]. The corresponding mechanical cost of transport (4) can then be calculated as *C* = 1.2*v*^2^/(*gl*_0_)sin(*φ*_0_)cos^2^(*φ*_0_). It should be noted that, due to the pure pendular motion, the power outputs during the motion, $F\dot{l}$ and $M\dot{\varphi }$, are always zero, and that the energy exchange is instantaneous at heel strike and toe off. The values of the energy loss due to impact and the corresponding mechanical cost of transport for the parameter values as given above, are presented in Table 1.

**Table 1:** Synthetic gait energy balance per step and cost of transport.

gait	Positive work during motion (J)	Energy loss during impact (J)	Cost of Transport (-)
pendular	0	64.80	0.11
level	127.64	0	0.22
sinusoidal	109.15	0	0.19

#### Level motion

**Fig 6:**
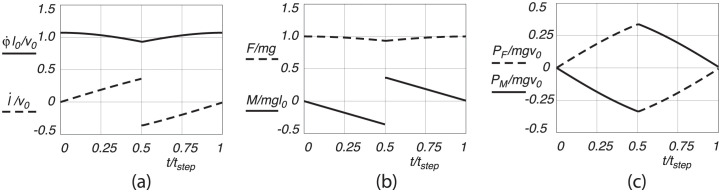
Level walking motion, with (a) dimensionless leg extension velocity $\dot{l}/v_{0}$ and pendular velocity $\dot{\varphi }l_{0}/v_{0}$, (b) dimensionless leg extension force *F*/(*mg*) and pendular motion force *M*(*mgl*_0_), and (c) dimensionless applied leg extension power *P*_*F*_/(*mgv*_0_) and pendular motion power *P*_*M*_/(*mgv*_0_), as a function of dimensionless time *t*/*t*_*step*_, for one step from midstance to midstance.

For the level motion we assume that the CoM moves purely horizontal at a constant forward speed. To maintain this constant height of the CoM, the stance leg has to extend and retract, see Fig 6a. In order not to accelerate during the motion, a torque *M* (or shear force *S* = *M*/*l*, perpendicular to the leg extension force *F*) has to be applied to the leg with ever increasing value until heel strike, see Fig 6b. Note that the force along the leg, *F*, supporting the mass remains almost constant. Since both forces and velocities are non-zero, power is applied to the system as shown in Fig 6c. The power output of the leg extension, *P*_*F*_ is positive in the first half of the motion from midstance to heel strike in order to support the weight during extension, whereas the power in the pendular motion, *P*_*M*_, is negative in order to decelerate the pendular motion. The deceleration is needed so that there is no impact at heel strike and toe-off. It can also be seen that most of the work ( = ∫ Pdt) is done around heel strike and toe-off. The values of the positive work done during the motion and the corresponding mechanical cost of transport, for the parameter values as given above are presented in Table 1.

#### Sinusoidal motion

**Fig 7:**
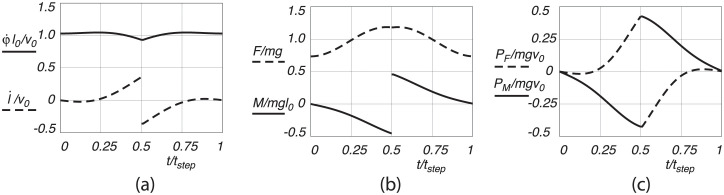
Sinusoidal walking motion, with (a) dimensionless leg extension velocity $\dot{l}/v_{0}$ and pendular velocity $\dot{\varphi }l_{0}/v_{0}$, (b) dimensionless leg extension force *F*/(*mg*) and pendular motion force *M*(*mgl*_0_), and (c) dimensionless applied leg extension power *P*_*F*_/(*mgv*_0_) and pendular motion power *P*_*M*_/(*mgv*_0_), as a function of dimensionless time *t*/*t*_*step*_, for one step from midstance to midstance.

In the sinusoidal motion, it is assumed that the CoM follows a sinusoidal path with constant forward speed. This pattern more closely resembles CoM motion in actual human gait, and fits between the pendular motion of arcs connected by cusps and the pure level motion. The leg extension from midstance to heel strike is postponed, and it even starts with a small negative value, see Fig 7a. Likewise, when considering the forces, most of the action of the pendular torque *M*, happens before the heel strike, whereas the leg force oscillates around the weight support, see Fig 7b. With the leg extension positive from midstance to heel strike, the leg power output, see Fig 7c, is positive and is concentrated towards the heel strike and toe-off. Likewise, the pendular torque *M*, to maintain constant speed and circumvent impact losses at heel strike, is also concentrated around the heel strike and toe-off. The values of the positive work done during the motion and the corresponding mechanical cost of transport for the parameter values as given above, are presented in Table 1. It can be seen that less work per step is involved compared to level walking. The mechanical cost of transport is less accordingly but still more than in the case of pendular walking.

### Experimental data

**Fig 8:**
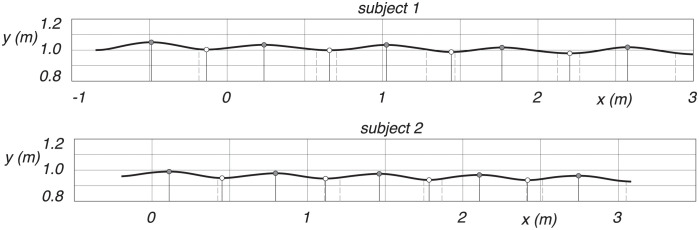
Measured CoM trajectories for subject 1 and 2, where *x* is forward, and *y* is vertically upwards. Midstance is indicated by a gray dot at the *y* maxima, and the discrete change from swing leg to stance leg is marked by a white dot at the *y* minima. Heel strike is the dashed vertical line before and toe-off the dashed vertical line after the discrete change from swing leg to stance leg.

The measured CoM trajectories of two subjects are studied in this section. We define the midstance position, where the leg is vertical in the local maxima of the trajectory, and the midstance is then used to define also the step length, it being the distance between two consecutive mid stances. The discrete switching from swing leg to stance leg is defined at the local minima of the trajectory. The double stance phase, starting with the heel strike of the swing leg and ending with the toe-off of the hind leg, is captured from the marker data. We expect the defined discrete switching to be between this heel strike and toe off. The two trajectories of the CoM, as seen in Fig 8, captures each five distinct midstance positions and four switchings.

**Fig 9:**
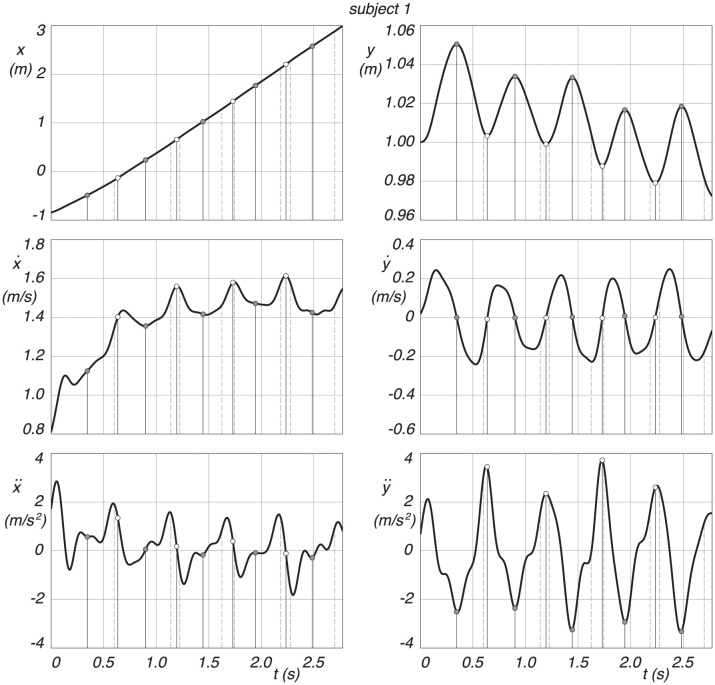
Position, velocity and acceleration of the CoM coordinates as a function of time, where *x* is forward and *y* is vertically upward, for subject 1. Midstance is indicated by a gray dot at the *y* maxima, and the discrete change from swing leg to stance leg is indicated by a white dot at the *y* minima. Heel strike is the dashed vertical line before and toe-off the dashed vertical line after the discrete change from swing leg to stance leg.

**Fig 10:**
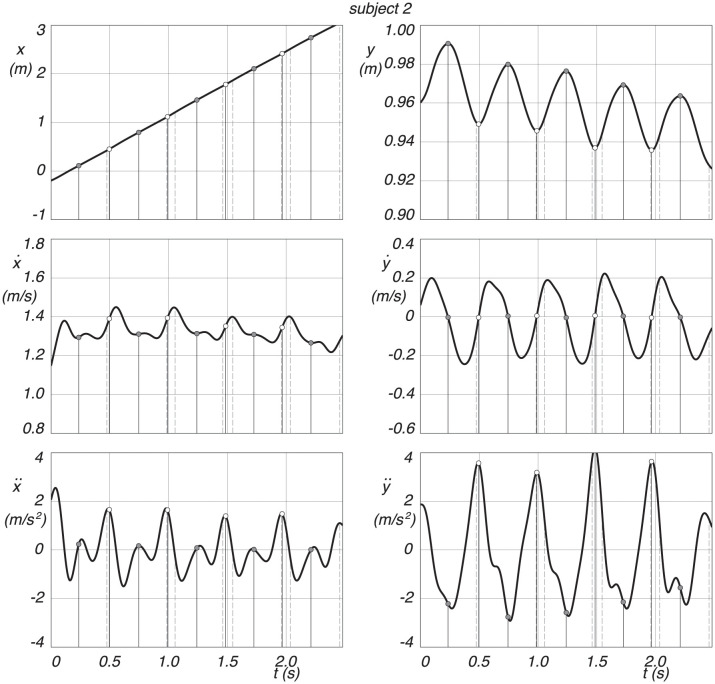
Position, velocity and acceleration of the CoM coordinates as a function of time, where *x* is forward and *y* is vertical up, for subject 2. Midstance is indicated by a gray dot at the *y* maxima, and the discrete change from swing leg to stance leg is indicated by a white dot at the *y* minima. Heel strike is the dashed vertical line before and toe-off the dashed vertical line after the discrete change from swing leg to stance leg.

The individual *x* and *y* coordinates of the CoM trajectory, together with their velocities and accelerations, for subject 1, can be seen in Fig 9. The results for subject 2 are shown in Fig 10.

**Fig 11:**
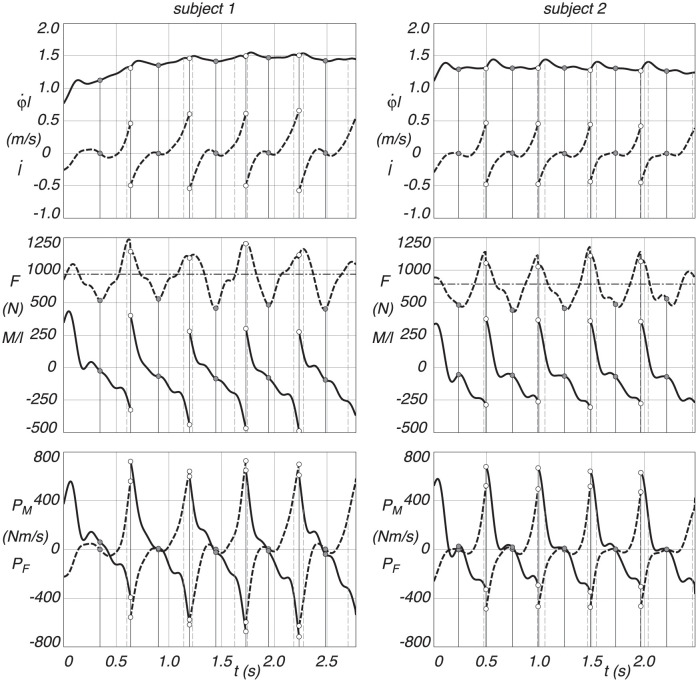
Tangential pendulum velocity $\dot{\varphi }l$ (solid line) and leg extension velocity $\dot{l}$ (dashed line), pendulum torque force *M*/*l* (solid line) and leg extension force *F* (dashed line), and applied pendular motion power *P*_*M*_ (solid line) and applied leg extension power *P*_*F*_ (dashed line), as a function of time, for Subject 1 and 2. The straight horizontal dash-dotted line at the leg extension force is the static gravity force *mg*. Midstance is indicated by a dash-dot vertical line, and the discrete change from swing leg to stance leg is indicated by a dotted vertical line, where the heel strike is the dashed vertical line before and the toe-off the dashed vertical line after a dotted vertical line.

For subject 1 and 2, the velocity of the leg extension, $\dot{l}$, and the tangential velocity, $\dot{\varphi }l$, of the CoM across five stances, are shown in Fig 11. After some initial transient, there is a clear periodicity in both velocities. The leg extension velocity clearly changes from positive to negative at the discrete switch from heel strike to toe-off, indicated by the dotted lines, whereas the pendular velocity oscillates around the average forward speed.

The constraint forces, *F* and *M*/*l*, driving the motion, are also shown in Fig 11. Clearly, the leg extension force *F* oscillates around the force of gravity for static support, where the minima are around mid stance, indicated by the dash-dot lines, and the maxima at heel strike and toe off, indicated by the dotted lines. The torque *M*, moving the pendulum motion, clearly shows extreme values at the discrete switch from heel strike to toe off and is around zero at mid stance. We see a negative torque at heel strike and a positive one at toe off, fighting gravity to keep a constant forward velocity.

The applied power for the leg extension, *P*_*F*_, and for the pendular motion, *P*_*M*_, are also shown in Fig 11. Most of the work (*W* = ∫ Pdt) is done around the discrete switch from heel strike to toe-off. Before the switch, one could say around heel strike, the leg extension is doing positive work by lifting the CoM from the pendular arc, whereas the pendular motion is doing negative work by fighting gravity to keep a constant forward velocity. After the switch, i.e., around the toe-off, the leg is contracting and doing negative work to keep the CoM level during the pendular motion, whereas the pendular motion is doing positive work by fighting gravity to keep a constant forward velocity. Note that subject 2, who has a shorter leg length and lower body mass than subject 1, shows a steadier cyclic walking motion compared to subject 1.

Table 2 presents a summary of the energy expenditure for the two subjects, detailing per step: step length, work done, and dimensionless mechanical cost of transport. For subject 1, who represents a gait pattern with longer step lengths and a higher body weight, the step length varies from 0.73 m to 0.81 m. Correspondingly, the work done ranges from 102.62 J to 127.70 J. The dimensionless cost of transport for this subject ranges from 0.15 to 0.17. Subject 2, on the other hand, represents a gait pattern with smaller step lengths at a lower body weight. The step length for this set ranges from 0.64 m to 0.68 m. The work done ranges from a minimum of 76.10 J to a maximum of 86.15 J, and the dimensionless cost of transport is between 0.14 and 0.15. Using the lowest cost of transport from Table 2 as a reference, for subject 1, the shortest step length, 2.4% shorter, shows a 1.11% increase in cost of transport, whereas a 9% longer step shows 13% higher cost of transport. Subject 2 has the lowest cost of transport at the shortest step, with the longest step, 7.4% longer, showing a 5.5% increase in the cost of transport. Additionally, our study further explored the effect of variation in vertical CoM displacement. An increase of 10% in the vertical CoM displacement shows a decrease of 10% in the cost of transport whereas 14% increase in cost of transport is observed for 10% decrease in vertical CoM estimate.

**Table 2:** Energy expenditure and step length per step for the two subjects.

Measurement	Step length (m)	Work done (J)	Dimensionless mechanical cost of transport (-)
subject 1	0.73	102.62	0.15
	0.79	120.78	0.17
	0.74	103.99	0.15
	0.81	127.70	0.17
subject 2	0.68	86.15	0.15
	0.67	84.63	0.15
	0.64	80.04	0.15
	0.64	76.10	0.14

**Fig 12:**
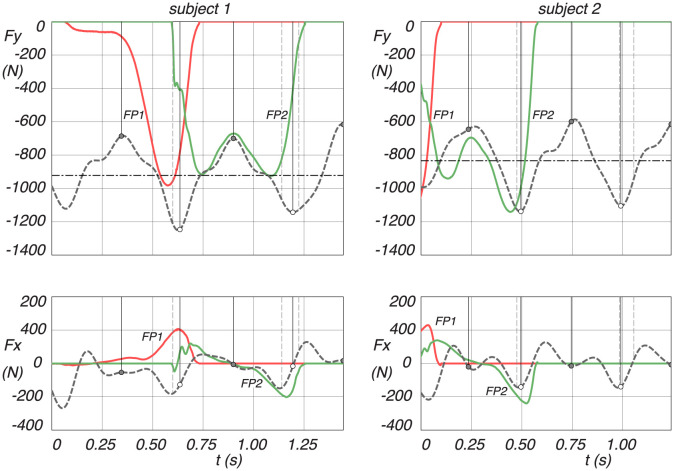
Measured ground reaction forces from force plate 1 (FP1 red solid lines) and force plate 2 (FP2 green solid lines) together with the ground reaction forces calculated from the model, *F*_*x*_ and *F*_*y*_, where *x* is forward and *y* vertically upwards. Midstance is indicated by a dash-dot line, and the discrete change from swing leg to stance leg is indicated by a dotted line, where the heel strike is the dashed line before and the toe-off the dashed line after a dotted line.

As a last result we present a comparison of the calculated ground reaction forces, *F*_*x*_ and *F*_*y*_, calculated from the model and the measured ground reaction forces, as shown in Fig 12. These forces were measured by two force plates, each capturing one stance leg during the walking motion. Unfortunately, force plate 1 (FP1) had some technical problems and only record properly after some initial milliseconds. For force plate 2 (FP2), it can be seen that the calculated data from the model follows the measured data pretty well. It is important to note that the force plate measurements serve primarily as an additional check, affirming that the model reflects realistic ground reaction forces in walking.

## Discussion

The analysis of synthetic walking data illustrates the energetics and efficiency of different gait patterns. In this study, the pendular motion, demonstrates the lowest cost of transport of the patterns studied. This result can be attributed to its pure projectile-like motion and the instantaneous work done during heel strike and toe-off. The pendular gait is associated with minimal changes in leg length and negligible knee flexion, which may result in reduced muscle activation and mechanical work compared to other gait patterns. However, the instantaneous work done during heel strike and toe off is unrealistic. Conversely, level walking shows the highest value for the cost of transport, aligning with the findings of [25] which revealed minimizing vertical displacement of the center of mass leads to higher metabolic costs due to increased mechanical work at the hip, knee and ankle joints. The sinusoidal motion, which represents a more natural gait pattern, exhibits a cost of transport between that of the pendular walking and level walking.

When applying the model to the measured data, we find a dimensionless mechanical cost of transport value between 0.14 and 0.17. This result aligns with findings from [26], where an average value of 0.2 at walking speeds of 1.25 m/s was recorded. It is worth noting that the cost of transport in [26] was calculated using the net metabolic rate—derived from oxygen consumption and carbon dioxide production rates—and normalized by gravitational force and velocity. Rehabilitation progress are often reflected by changes in metabolic cost of transport as in [27–29]. Furthermore, there is a clear correlation between metabolic and mechanical cost of transport as established in [8, 11, 15]. Our work proposes that the mechanical cost of transport as calculated by our simplified model, can be a representative indicator of the metabolic cost of transport, and monitoring this mechanical cost of transport could thus provide insights into rehabilitation progress in walking.

The approach suggested in this work allows the mechanical cost of transport per step to be analyzed, offering more detailed insights into energy expenditure during walking. The trajectory of the CoM in subjects with longer step lengths and higher body weight showed a higher energy expenditure, which could be attributed to the increased forces needed to move a larger mass over a more extended distance. Smaller step lengths and lower body weights required less work, but the efficiency, as indicated by the dimensionless mechanical cost of transport, was not significantly different between the two subjects. This finding suggests that while the magnitude of work done during walking might change with individual anthropometrics, the overall efficiency of the gait pattern is maintained across variations in step length and body weight. This characteristic underlines the potential for adopting a uniform algorithm to analyze gait across a diverse demographic.

## Conclusion

This study showed that using a simple mechanical model for human walking applied to the kinematic motion data of the center of a mass of the walking human, results in realistic predictions of the dimensionless mechanical cost of transport. Moreover, the model is able to calculate this mechanical cost of transport per step, making it possible to monitor progress in walking rehabilitation.

The findings open many interesting avenues. Future work will be directed towards obtaining the kinematic motion data of the center of mass of a human during walking by means of one simple inertial measurement unit. This would open the way to remote and off-line monitoring of the rehabilitation progress.
